# Adequacy of ovarian diathermy under ultrasound control: an experimental model

**DOI:** 10.1186/1757-2215-6-54

**Published:** 2013-07-25

**Authors:** Anita Mylius Pimentel, Lucia Maria Kliemann, Daniela dos Santos Brum, Fábio Gallas Leivas, Paulo Roberto Stefani Sanches, Edison Capp, Helena von Eye Corleta

**Affiliations:** 1 Programa de Pós-Graduação em Medicina: Ciências Médicas, Hospital de Clínicas de Porto Alegre, Universidade Federal do Rio Grande do Sul, Porto Alegre, Brazil; 2 Departamento de Patologia, Faculdade de Medicina, Hospital de Clínicas de Porto Alegre, Universidade Federal do Rio Grande do Sul, Porto Alegre, Brazil; 3 Departamento de Ginecologia e Obstetrícia, Faculdade de Medicina, Hospital de Clínicas de Porto Alegre, Universidade Federal do Rio Grande do Sul, Porto Alegre, Brazil; 4 Laboratório de Biotecnologia da Reprodução (Biotech), Campus Uruguaiana, Universidade Federal do Pampa (UNIPAMPA), Uruguaiana, Brazil; 5 Serviço de Pesquisa e Desenvolvimento em Engenharia Biomédica-GPPG, Centro de Pesquisas, Hospital de Clinicas de Porto Alegre, Universidade Federal do Rio Grande do Sul, Porto Alegre, Brazil; 6 Serviço de Ginecologia e Obstetrícia - Hospital de Clínicas de Porto Alegre, Rua Ramiro Barcelos, 2350/11º andar, Porto Alegre, RS CEP 90035-903, Brazil

**Keywords:** Transvaginal ovarian drilling, Ovarian diathermy, Bovine, Polycystic ovary syndrome

## Abstract

**Background:**

To develop a minimally invasive ovarian cauterization technique under transvaginal ultrasound control and evaluate the safety and feasability of monopolar cauterization to cause ovarian injury using female cattle of reproductive age as an experimental model.

**Method:**

Experimental study in a university research center was performed. Eleven female bovines of reproductive age were submitted to monopolar transvaginal ovarian cauterization. The right ovary (RO) was punctured at four sites and 40 W was applied for 5 s at each point, resulting in a total of 800 J (Joules) of thermal energy. In the left ovary (LO), the procedure was similar, with the same time and 80 W, resulting in a thermal energy of 1600 J. Macroscopic and microscopic lesions were assessed.

**Results:**

Of 22 ovaries punctured, 20 were cauterized and exhibited macroscopic and typical microscopic lesions. No lesions could be found in the needle path. The measures of the areas of microscopic electrocautery lesions calculated estimating a cylindrical volume showed a median of 1.12% in the right ovary and 1.65% in the left ovary. When the estimate was calculated by spherical shape, the medians were 1.77% in the right ovary and 3.06% in the left ovary. There was a statistically significant difference in these two estimates (sphere, p = 0.008; cylinder, p = 0.021).

**Conclusion:**

The experimental animal model described for transvaginal ultrasound-guided ovarian needle cauterization seems to be feasible. The ovaries were successfully cauterized without injuries in needle path and more energy resulted in significantly more thermal lesion. The safety and effectiveness of this technique, theoretically less invasive than current ovarian drilling methods, could be tested in anovulatory women with PCOS.

## Introduction

Polycystic ovary syndrome (PCOS) is a multifactorial syndrome characterized by at least two of the following three changes: anovulation, hyperandrogenism, and enlarged ovaries on ultrasound [1,2]. Due to its high prevalence, it is the most common cause of anovulatory infertility. The latest consensus on treatment of PCOS-related infertility, published in 2008, advocates the use of clomiphene citrate (CC) as first-line treatment for ovulation induction [3,4]. However, around 20% of PCOS patients are resistant to CC, requiring second-line intervention—exogenous gonadotrophins or surgical induction (ovarian diathermy)—to ovulate [3]. After 6 months of treatment, both interventions have equal pregnancy rates [5]. Ovulation induction with gonadotropins requires intense monitoring of ovarian response, and is associated with increased occurrence of multiple pregnancy and ovarian hyperstimulation syndrome (OHSS) [6].

Laparoscopic ovarian diathermy (LOD) is associated with high rates of ovulation (80% to 90%) and pregnancy (60% to 80%) [5,7,8]. A single LOD procedure leads to repeated ovulatory cycles and potential pregnancies without the need of repeated drug treatments; it is important to induce mono-ovulation, without the risk of multiple pregnancy [9].

Its advantages notwithstanding, LOD is an inpatient treatment, requires general anesthesia, and the risk of postoperative adhesions cannot be ignored [8,10]. Seeking to reduce adhesions and simplify ovarian cauterization, microlaparoscopy without general anesthesia [11] and laser-based transvaginal ovarian drilling (TVOD) techniques have been described [11-14].

A brief communication from Syritsa *et al.* in 1998 reported their results of transvaginal ovarian electrocoagulation in 6 anovulatory patients with PCOS. There were no complications, and after 8 weeks, 4 patients were pregnant [15].

To test the efficacy and safety of ultrasound-guided TVOD, we are pursuing an animal experimental model. Using sheep as an experimental model [16], there were no intrapelvic thermal injuries secondary to transvaginal cauterization; however, effectiveness could not be demonstrated. The aim of this study was to establish the feasibility and safety of transvaginal ultrasound ovarian diathermy with monopolar cauterization in a different animal model (breeding-age cows).

## Materials and methods

**Figure 1:**
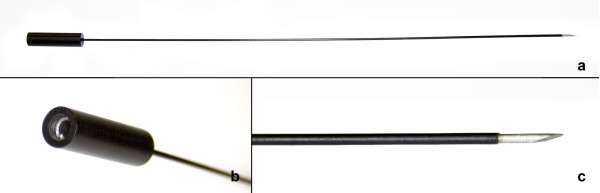
**Needle developed for transvaginal ovarian cauterization. (a)** The needle proper; **(b)** electrocautery connector; **(c)** non-insulated distal tip.

The study sample comprised 11 female cattle, aged 2–3 years. Two days before slaughter, cows underwent ovarian cauterization. The cauterization needle (stainless steel, diameter 1 mm and length 55 cm, insulated throughout its length except for the distal 3 mm) was designed exclusively for TVOD by Helena von Eye Corleta and manufactured at the Department of Biomedical Engineering, Hospital de Clínicas de Porto Alegre. The proximal end of the needle was connected to the electrocautery device (Figure 1).

On the day of the experiment the animals were immobilized, and acepromazine 1% was administered for sedation and 2% lidocaine for low epidural anesthesia. In the vaginal probe, a guide was attached and the puncture needle was inserted through this guide. The ultrasound equipment used was an Aloka 500 (Aloka, Tokyo, Japan) with vaginal probe of 5 MHz (UST9111).

After identification of the ovaries, sonographic measurements (length, width and thickness, in centimeters) were obtained and then adjusted by the classical formula for a prolate ellipsoid (V = L × W × T × 0.523) [17].

The right ovary (RO) was punctured at four sites and 40 W was applied for 5 s at each point, resulting in a total of 800 J (Joules) of thermal energy. In the left ovary (LO), the procedure was similar, with the same time and 80 W, resulting in a thermal energy of 1600 J. The electrocautery was a Valleylab Force FX device with monopolar coagulation (Valleylab, Boulder, USA). The amount of energy was defined by the work of Amer *et al.*[10], which showed that 3 to 6 punches (450 to 900 joules) per ovary result in rates significantly better for ovulation and pregnancy in women with PCOS.

Two days after the procedure, the cows were slaughtered and macroscopic examination of the ovaries was performed with a thorough inspection of the needle path, looking for lesions secondary to cauterization or puncture. Immediately afterwards, the ovaries were collected and their volumes measured by the displacement method in a beaker of water. The ovaries were fixed in 10% formalin and histological analysis was performed by experienced pathologist and changes caused by the puncture and cauterization were identified.

After 10 days in formalin, the ovaries were measured with a ruler (height, length and width in centimeters) and volume calculated with the aforementioned formula (V = L × W × T × 0.523) [17].

Macroscopic analysis was performed by the pathologist and serial sections (3 mm thick) were obtained from each specimen. The puncture site and any anomalous areas were identified, and the ovaries were sent for microscopic examination to control for normal ovarian parenchyma. All specimens underwent routine histological processing (dehydration in alcohol, clarification in xylene and paraffin embedding). Blocks were sliced into 4 μm-thick sections, on two planes spaced 50 μm apart, and stained with hematoxylin and eosin. Microscopic analysis sought to assess the effects caused by the electric current and the temperature change due to the different power applied. Microscopic analysis was also performed by a pathologist without knowledge of ovary laterality.

Ovarian lesion area was quantified using the ImageJ 1.40G software suite (Wayne Rasband, National Institutes of Health, Washington, D.C., USA, public domain). Slides were reviewed, the largest lesion in each ovary was quantified by ImageJ, and lesion area was estimated as part of a cylinder or a sphere. To calculate the first approximation, the lesion was estimated to have a uniform thickness of 3 mm because, as the needle has a diameter of 1 mm, we estimated 100% lesion extension for each side with a cylinder-shaped injury, as described elsewhere [18]. To calculate the second approximation, the lesion was estimated to have the volume of a sphere (V = 4/3*π*r^3^) whose radius is equal to the square root of the area calculated in ImageJ over π (r = √A/π).

To define the percentage of lesions in each ovary, we divided the lesion volume defined by these two approaches (cylindrical and spherical) by the ovarian volume estimated by water displacement.

We performed the Shapiro-Wilk test with Lilliefors correction for review of normal results. As the results did not show a normal distribution, the Wilcoxon signed-rank test was used to compare the variables (both features as outcomes) between the right ovary (800 J) and left (1600 J). The Friedman test was used to compare ovarian volume at three different points in time (at ultrasound, after slaughter and after formalin). The correlation between ovarian volumes obtained by ultrasound, after slaughter and after formalin was evaluated by the Spearman correlation coefficient. The significance level for all tests was set at P ≤ 0.05. All analyses were carried out in SPSS 19.0 (*Statistical Package for the Social Sciences*).

This experiment was performed in accordance with Brazilian College of Animal Experimentation (*Colégio Brasileiro de Experimentação Animal*, COBEA) guidelines and was approved by the Hospital de Clínicas de Porto Alegre Research Ethics Committee (#110072).

## Results

Ovarian cauterization was performed in 11 female cattle (age 2–3 years, mean weight 466 ± 23.3 kg). The sonographic characteristics of these ovaries and their volume—measured by ultrasound, on the day of slaughter, and after 10 days in 10% formalin—are shown in Table 1. Of the right ovaries, five had 1 follicle larger than 10 mm, one had 2 follicles, and 6 had a *corpus luteum*. Of the left ovaries, four had 1 follicle larger than 10 mm, four had 2 follicles larger than 10 mm and four had a *corpus luteum*. The characteristics of right and left ovaries were similar, thus enabling comparison of the two groups after the use of different power settings for ovarian cauterization (Table 1).

**Table 1:** Characteristics of the ovaries obtained from 11 cows

	**Right ovary (SD)**	**Left ovary (SD)**
Volume at ultrasound (cm^3^)	27.79 (12.62)^**a**^	19.51 (6.21)^**b**^
Volume after slaughter (cm^3^)	10.27 (6.3)	11.45 (4.8)
Volume after formalin (cm^3^)	8.25 (5.75)	8.03 (5.18)
Presence of follicles > 10 mm (n)	7	12
Presence of corpus luteum (n)	6	4

There were 11 ovaries of each side at the day of the procedure (ultrasound) and 10 ovaries of each side after slaughter. Volume at ultrasound was statistically different from the other two volumes measured (^**a**^p = 0.003; ^**b**^p < 0.001).

**Figure 2:**
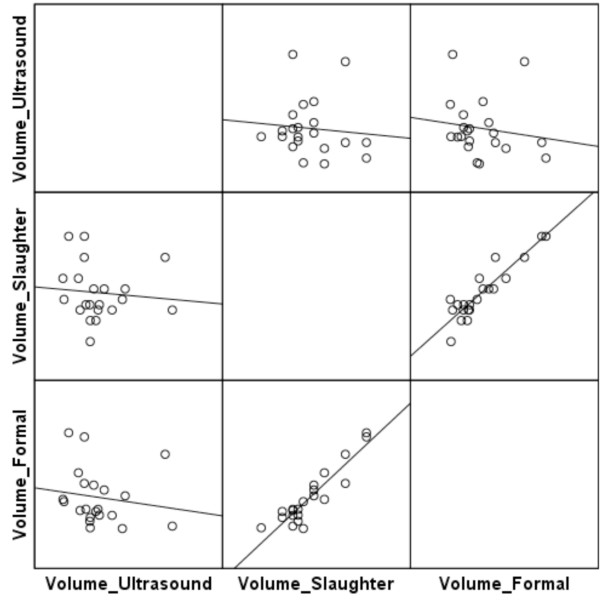
**Correlation of ovarian volumes after ultrasound, after slaughter and after formalin.** Correlation coefficient = 0.841.

The correlation between the different methods for determination of ovarian volumes is shown in Figure 2. There was a strong correlation (r = 0.841) between ovarian volumes after slaughter (measured by volume displacement of water) and after formalin. The volume estimated by ultrasound did not correlate with other measures, being significantly higher (p = 0.003).

**Figure 3:**
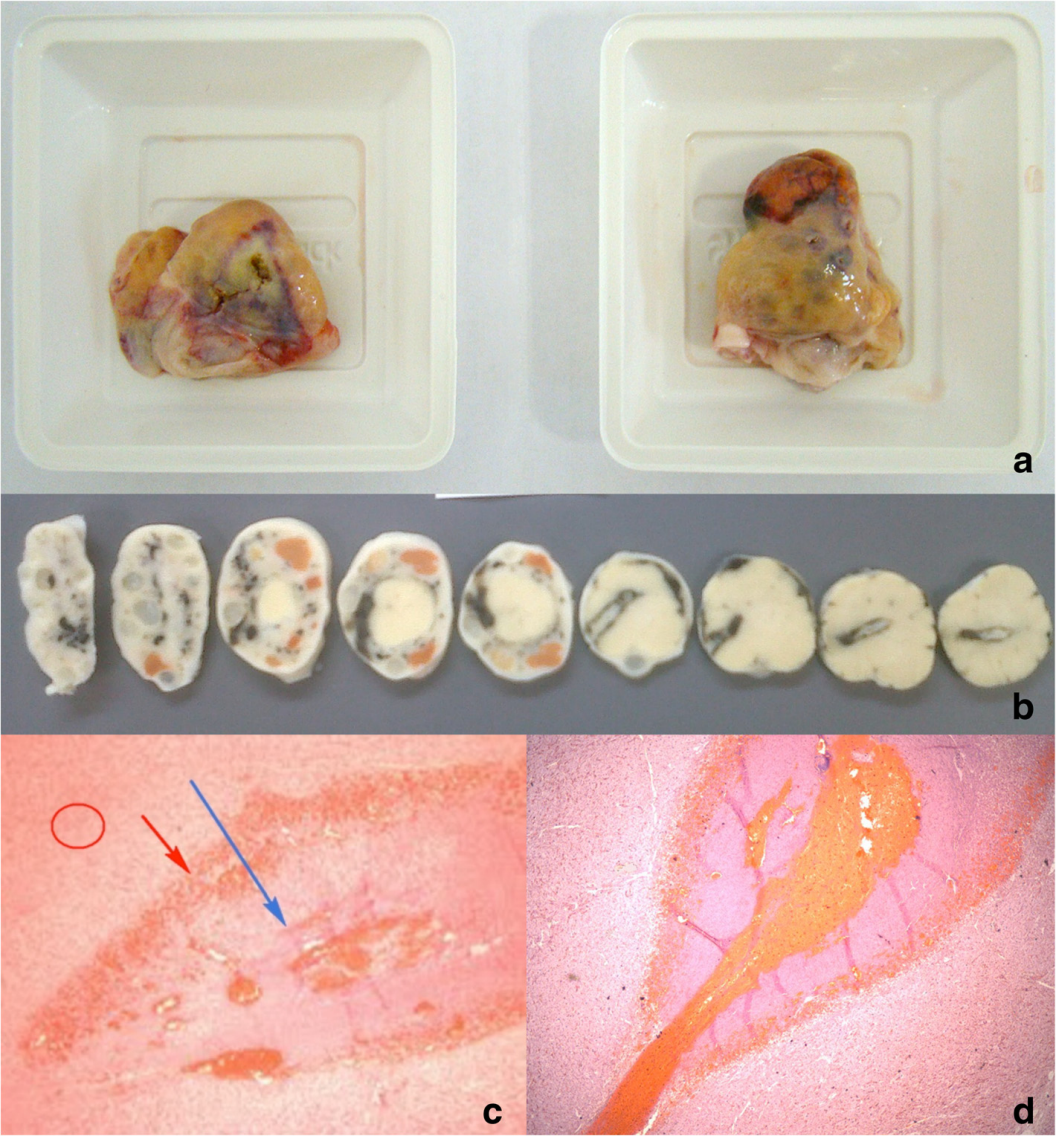
**Macroscopy and histology of the ovaries. (a)** ovaries of animal 6333 after slaughter, showing macroscopic lesions in the left and right ovaries; **(b)** ovarian sections of animal 6333 after formalin; **(c)** SB2 right ovary, histology (40× magnification), after hematoxylin-eosin staining – red circle, normal ovary; red arrow, hemorrhagic area at the lesion border; blue arrow, center of the lesion with coagulation necrosis; **(d)** 6333 right ovary, histology (40× magnification), after hematoxylin-eosin staining.

Of 22 cauterized ovaries, only two had no visible lesions, due to problems during ovarian collection at the slaughterhouse where these two ovaries were sectioned, with only a small segment of each left for examination. The LO of cow SB1RA and RO of cow 6351 were sectioned and no lesions could be identified in the remaining segments. Although the total thermal dose delivered was 800 J (4 × 5 s × 40 W) on the right side and 1600 J (4 × 5 s × 80 W) on the left, no lesion could be found along the needle path. The other 20 ovaries exhibited macroscopic and histological lesions as expected (Figure 3). The characteristic microscopic histological lesion seen 2 days after cauterization is identified as hemorrhage, necrosis and perivascular neutrophil infiltration [16,19,20].

**Figure 4:**
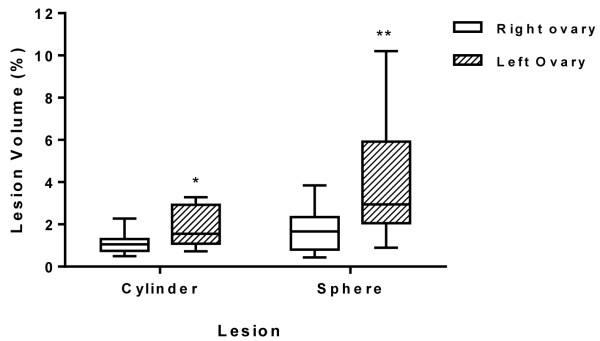
**Box-and-whisker plot of mean ovarian tissue damage.** Lesions estimated by cylinder or sphere volume. The *upper line* of the box represents the upper quartile, the *thick line* in the middle represents the median, and the *lower line* represents the lower quartile. Lesion volumes estimated by the two methods were statistically different between the two ovaries (*p = 0.021 and **p = 0.008).

The measures of the lesioned areas calculated by cylindrical volume estimates showed a median of 1.12% in the right ovary and 1.65% in the left ovary. When estimation was calculated by spherical shape, the medians were 1.77% in the right ovary and 3.06% in the left ovary. There was a statistically significant difference in these two estimates of lesion area of the right ovaries (800 J) and left ovaries (1600 J) (p <0.05), as shown in Figure 4.

## Discussion

Ultrasound-guided TVOD proved effective and safe in this experimental model. Cows are a suitable experimental model due to the similarities between their ovary size and location in the vaginal vault and estrous cycles and those of human females [18,21]. All animals subjected to the procedure showed typical macroscopic and microscopic lesions due to monopolar ovarian cauterization, with no lesions found in the needle path.

Ovarian volumes measured by ultrasound were quite different from those measured using the other two methods. This finding is compatible with that reported by Brett *et al.*, who demonstrated a lack of precision in ovarian volume measurement by 2D and 3D ultrasound, which overestimate ovarian volumes [22]. The authors advise that the possibility of error should be taken into account when making clinical decisions. The ovarian volumes measured after slaughter and after formalin were correlated, as expected.

Only two previous studies have reported the amount of histological damage after ovarian drilling procedures. El-Sheikhan *et al.* (2004) demonstrated lesions in 0.38 to 1% of the ovary (in 42-to-45-year-old patients without PCOS) using 4 (800 J) and 8 (1600 J) punctures at a power setting of 40 W over 5 seconds and immediate removal after drilling [20]. Hendriks *et al.* compared the extent and characteristics of *in vitro* tissue damage in the bovine ovary after bipolar, monopolar or carbon dioxide (CO_2_) laser ovarian drilling [18]. Bipolar electrocoagulation resulted in significantly more ovarian destruction than CO_2_ laser and monopolar electrocoagulation. The damage induced by CO_2_ laser and monopolar coagulation was approximately 0.2–1.0 cm^3^[18]. These results are not comparable to those of the present study, as the energy settings were not comparable and the Hendriks *et al.* study was conducted *in vitro*. Tissue vascularization probably results in less damage than that observed *in vitro*, as differences in water content and the heat sink effect might mitigate injury [23].

This is the first publication addressing *in vivo* ovarian damage 48 h after drilling. The extent of damage in the right ovary (800 J) ranged from 1.12 to 1.77%, and in the left ovary (1600 J), from 1.65 to 3.06%. Smaller bovine ovaries, comparable to ovaries from women above the age of 40 years [20], could be more resistant to thermal damage than the soft, large, micro- and macrocyst-filled polycystic ovaries of younger women. Inflammatory response and tissue reaction 48 h after drilling, even with 1600 J, affected approximately 5% of the cow ovary. As patients with polycystic ovary syndrome have an increased ovarian volume (>10 cm^3^), the extent of ovarian injury is expected to be even less, especially with the use of non-aggressive protocols (<800 J) as proposed by Amer *et al.*[10]. This amount of destruction will not cause premature ovarian failure, which is one of the concerns associated with diathermy-based ovarian drilling [24].

Another concern is the presence of periovarian adhesions after drilling. Mercorio *et al.* described a high incidence of adhesion formation after LOD on second-look laparoscopy [25]. Hendriks *et al.* demonstrated that the cylinder-shaped lesion of monopolar electrocoagulation reduces ovarian surface injury as compared with the cone-shaped lesions of laser drilling [18]. It is believed that the greater the ovarian surface damage, the higher the risk of periovarian adhesions [18]. On the other hand, Taskin *et al.* associated adhesions in LOD with CO_2_ pneumoperitoneum [11], which is not required in TVOD.

Intraoperative complications of laparoscopic drilling involve the common complications of laparoscopy or of general anaesthesia [26], which can be minimized with the transvaginal approach. There is also risk of an electrical accident, that can be avoided by introducing all the not insulated extremity in the ovarian tissue, procedure that an experienced ultrassonographist in oocytes recovery performs with safety. Regarding the incidence of ovarian failure after the procedure, it seems that substantial reduction in the number of pre-antral and antral follicles in patients with PCOS does not necessarily lead to a diminished ovarian reserve. Only one case report has been published reporting ovarian atrophy after ovarian drilling, which was probably related to disruption of the blood supply [26]. Weerakiet *et al.* studied the ovarian reserve in patients with PCOS after drilling, and found to be lower than in nonsurgical patients with PCOS, but greater than in age-matched non-PCOS controls [27]. The volume of ovarian damage in our monopolar drilling experiment reach less than 2% of total ovarian volume with 800 J, the amount of energy recommended by Amer [10], certainly this could no result in ovarian failure.

So far all not laparoscopic routes proposed the bipolar ovarian drilling, our study was the first that advocates the use of transvaginal monopolar energy. The group from Hendriks was the unique that evaluate quantitatively the extend of ovarian damage after mono and bipolar drilling and showed significantly more damage with bipolar energy [18]. Until more studies to evaluate the best method to perform drilling are done it is essential to use the monopolar electrocoagulation, the technique with the lowest possible ovarian damage.

TVOD performed in human PCOS patients with ovarian volumes in the region of 10 cm^3^, using the expertise and safety of transvaginal puncture for oocyte retrieval [28], should be easier than in cows, and would probably constitute a simple, feasible procedure, as suggested by Syritsa in 1998 [15].

## Conclusions

The bovine model described herein appears to be an appropriate experimental animal model for transvaginal ultrasound-guided needle ovarian cauterization. The absence of needle path lesions, the significantly greater termal microscopic histologic lesion produced with higher energy shows the feasibility of the proposed procedure. The safety and effectiveness of this less invasive monopolar drilling approach encourages its use for surgical ovulation induction in PCOS women.

## Competing interests

The material contained in the manuscript has not been published or submitted elsewhere and it has not any vested conflict.

## Authors’ contributions

AMP participated in the conception and design of study, data collection, data analysis and interpretation, surgery and imaging procedures, statistical analysis, manuscript preparation. LMK participated in the data collection, data analysis and interpretation, manuscript preparation. DSB participated in the data collection, data analysis and interpretation, surgery and imaging procedures. FGL participated in the data collection, data analysis and interpretation, surgery and imaging procedures. PRSS developed the needle, participated in the data analysis and preparation of the manuscript. EC participated in the conception and design of study, data analysis and interpretation, statistical analysis, manuscript preparation. HEC participated in the conception and design of study, data collection, data analysis and interpretation, surgery and imaging procedures, statistical analysis, manuscript preparation. All authors read and approved the final manuscript.
